# Feature selection of EEG signals in neuromarketing

**DOI:** 10.7717/peerj-cs.944

**Published:** 2022-04-26

**Authors:** Abeer Al-Nafjan

**Affiliations:** Computer Science, Al-Imam Mohamed Ibn Saud Islamic University, Riyadh, Saudi Arabia

**Keywords:** Neuromarketing, Brain computer interface (BCI), Electroencephalogram (EEG), Deep neural network (DNN), Consumer preferences

## Abstract

Brain–computer interface (BCI) technology uses electrophysiological (EEG) signals to detect user intent. Research on BCI has seen rapid advancement, with researchers proposing and implementing several signal processing and machine learning approaches for use in different contexts. BCI technology is also used in neuromarketing to study the brain’s responses to marketing stimuli. This study sought to detect two preference states (like and dislike) in EEG neuromarketing data using the proposed EEG-based consumer preference recognition system. This study investigated the role of feature selection in BCI to improve the accuracy of preference detection for neuromarketing. Several feature selection methods were used for benchmark testing in multiple BCI studies. Four feature selection approaches, namely, principal component analysis (PCA), minimum redundancy maximum relevance (mRMR), recursive feature elimination (RFE), and ReliefF, were used with five different classifiers: deep neural network (DNN), support vector machine (SVM), k-nearest neighbors (KNN), linear discriminant analysis (LDA), and random forest (RF). The four approaches were compared to evaluate the importance of feature selection. Moreover, the performance of classification algorithms was evaluated before and after feature selection. It was found that feature selection for EEG signals improves the performance of all classifiers.

## Introduction

Technology is transforming traditional marketing by personalizing strategies and operations and making them more immersive for consumers. It is creating ecosystems that are more integrated and targeted for consumers. Neuromarketing is the research area that studies how to measure, record, and analyze signals related to the physiological response of the brain as well as other organs to specific stimuli and in diverse market contexts. It is a subfield of organizational cognitive neuroscience (Lee, Butler & Senior, 2010).

Brain–computer interface (BCI) is used as a consumer neuroscience tool that studies consumer preferences and behaviors in different marketing contexts. It is challenging for researchers and data analysts to handle high-dimensional electrophysiological (EEG) data in the field of machine learning. Feature selection provides an effective solution to handle this problem. Feature selection optimizes the selection of features and reduces feature size, decreasing the computation time and enhancing the learning precision (Cai et al., 2018).

In EEG-based BCI, feature space is high dimensional; feature selection is important because it acquires the signal characteristics that best depict EEG signals to be labeled. Feature selection is not the same as dimensionality reduction although they both reduce the data’s features. Feature selection does not alter properties but eliminates some based on certain conditions (Torres et al., 2020).

After feature extraction, feature selection can be used to choose a subset of features and reduce the size of the data required by the classification module (Abdulkader, Atia & Mostafa, 2015). Feature selection aims to choose features that contribute most substantially to the outcome class. The importance of feature selection is shown in the following points by Torres et al. (2020):

- Certain features can be redundant or unrelated to the preference states. Choosing a limited number of features allows for the possibility of monitoring the features relevant to the targeted preference states from a knowledge extraction perspective. Less redundant data will decrease overfitting, reducing the possibility of making a prediction based on noise.
- The number of features chosen is related to the number of parameters required to optimize classification algorithms. As the number of selected features is reduced, the number of optimization parameters is also reduced, resulting in the decrease of overtraining, which yields rapid predictions that are computationally efficient with low storage requirements.
- Feature selection improves classification performance with less misleading data and high accuracy.

Many studies of BCI systems have demonstrated that combining several feature types (*e.g*., combining time instances with frequency powers) increases the classification accuracy and dimensionality. Hence, selecting the most relevant features is essential to reduce the computational overhead arising from dimensionality. To solve this issue, several feature selection methods used in BCI studies were selected for benchmark testing (Jiang et al., 2020; Jenke, Peer & Buss, 2014; Moon et al., 2013). Principal component analysis (PCA), ReliefF, minimum redundancy maximum relevance (mRMR), and recursive feature elimination (RFE) were used. These four approaches were compared to evaluate the importance of the selected features. The performance of five different classifiers, namely, deep neural network (DNN), support vector machine (SVM), k-nearest neighbors (KNN), linear discriminant analysis (LDA), and random forest (RF), before and after feature selection was also evaluated.

Recent research in neuromarketing, such as those conducted by Pei & Li (2021), Khurana et al. (2021), and Gill & Singh (2021), highlighted the importance of using a combination of EEG features to achieve better recognition of emotions for decision-making. These studies also emphasized the role of feature selection to avoid dimensionality and increase the training and testing speed in neuromarketing research. Concomitantly, they indicated the lack of research studies that provide details regarding EEG features selection topic. To the best of my knowledge, this is the first BCI-based neuromarketing research that evaluated the importance of EEG indices with different algorithms of feature selection and classification.

The remainder of this study is organized as follows. After discussing the background of feature selection, the fundamental methods used for feature selection in a BCI were explored and reviewed. Moreover, the author illustrates the methodology and reports and discuss the experimental results. Finally, the research conclusion is added.

## Background

**Figure 1:**
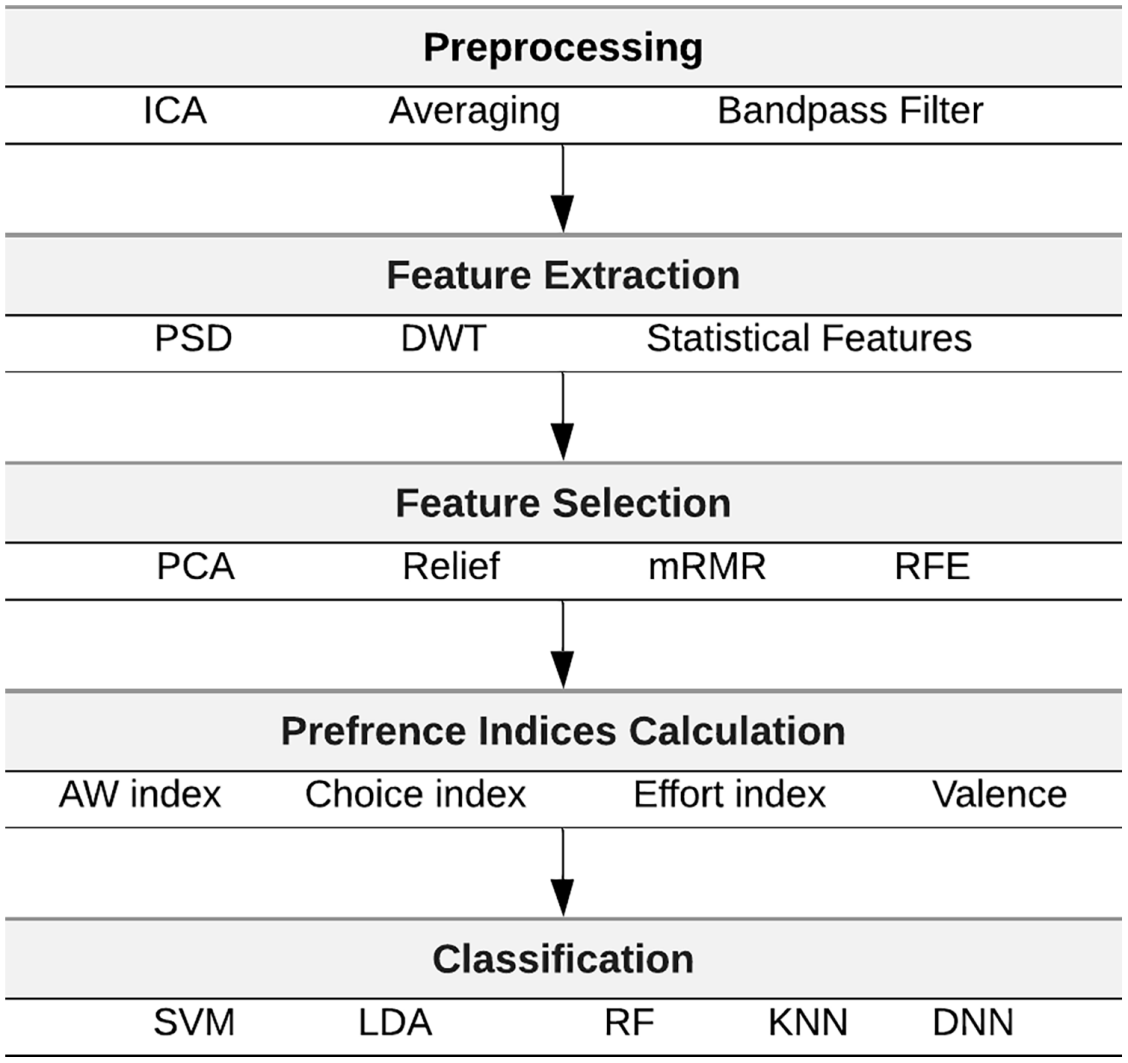
Architecture of the consumer preferences recognition system.

In Aldayel, Ykhlef & Al-Nafjan (2020b), EEG-based BCI was employed to predict consumer preferences and choices when viewing e-commerce products. Preference evaluation was performed in each trial to determine whether consumers liked or disliked the product and to test whether they would buy the product by recognizing preference patterns in consumer behavior. The proposed BCI system for preference detection comprises four key modules: signal preprocessing, feature extraction, feature selection, and classification. Figure 1 illustrates the methods used EEG-based consumer preference recognition system in Aldayel, Ykhlef & Al-Nafjan (2020a). In this section, a high-level description of feature extraction, feature selection, and preference indices key modules are presented.

### Feature extraction

**Figure 2:**
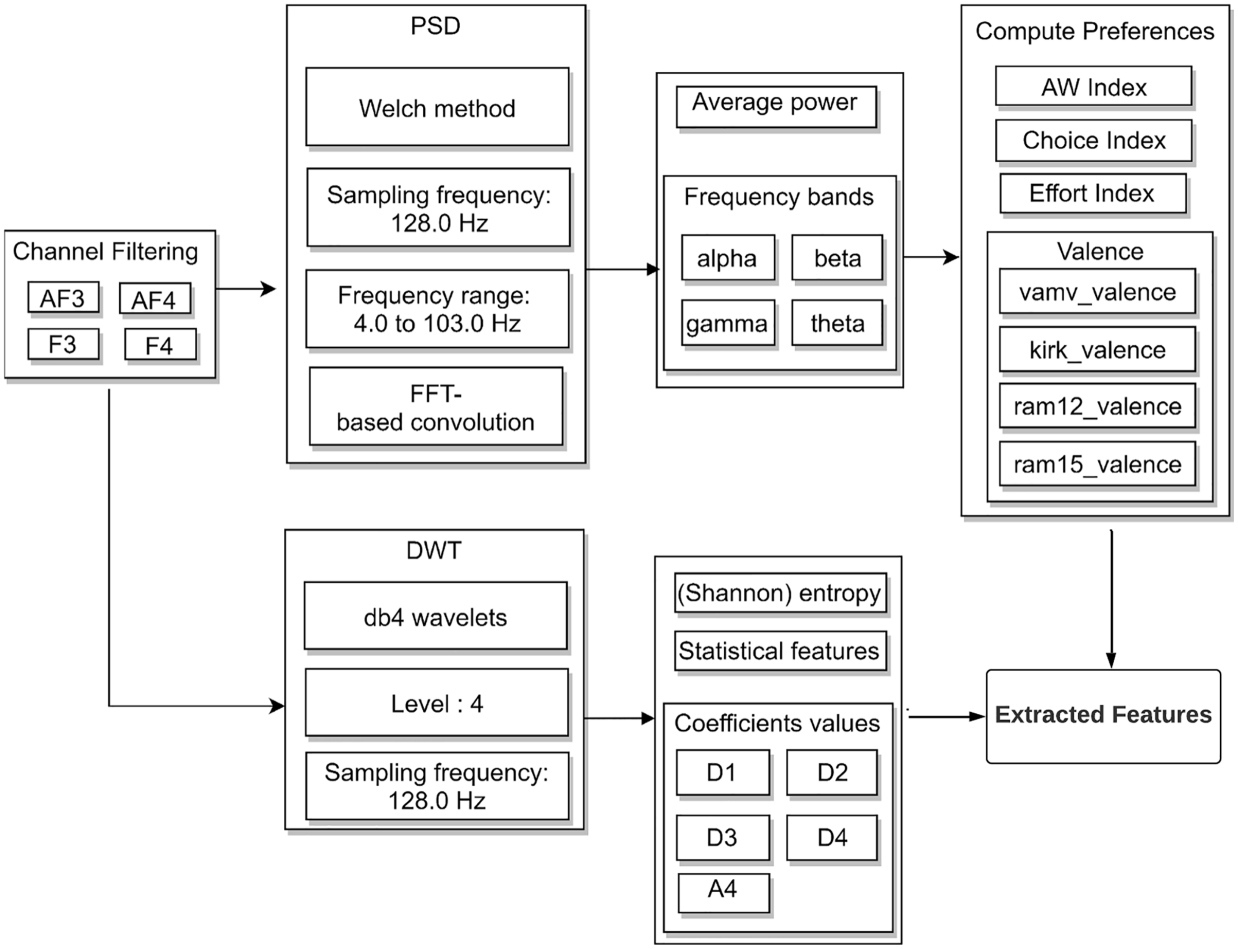
Feature extraction block diagram in neuromarketing dataset.

Feature extraction generates the discriminative features of the enhanced signals. In a previous research (Aldayel, Ykhlef & Al-Nafjan, 2021), the extraction of EEG features is described using two algorithms, *i.e*., discrete wavelet transform (DWT) and power spectral density (PSD). Figure 2 presents the block diagram of feature extraction.

EEG signals were filtered to electrodes (F3, F4, AF3, and AF4) that are relevant to preference recognition. In PSD, four frequency bands: theta (4–8 Hz), alpha (8–12 Hz), beta (12–30 Hz), and gamma (above 30 Hz) were extracted and the average signal power was calculated. In DWT, the EEG signals decomposed using four-level Daubechies (db4) wavelets into detail coefficients (D1, D2, D3, and D4) and an approximation coefficient (A4). The numbers of PSD and DWT features were 2,367 and 840, respectively. In this study, PSD is used for extracting features and the effect of feature selection in neuromarketing is evaluated.

### Feature selection

For BCI systems, the following fundamental feature selection approaches can be used: filtering, wrapper, embedded, and hybrid. Wrapper and embedded approaches use a classifier (baseline model) to select a subset of features, unlike filtering, which is model independent (Jenke, Peer & Buss, 2014).

- Filter approaches compute the level of association between each feature and the selected class independent of the classifier used. They can effectively detect irrelevant features, but they cannot eliminate redundancy or dependency between features. The main advantages of filter approaches are scalability, speed, and reduced computational overhead. Examples of filtering include maximum mutual information and mRMR, which is popularly used in EEG-based BCIs (Solorio-Fernández, Carrasco-Ochoa & Martínez-Trinidad, 2020; Jenke, Peer & Buss, 2014).
- Wrapper approaches evaluate the feature subsets using the results of a classification algorithm until the accuracy result is satisfied. They can achieve high accuracy, but due to the high computational cost and time, they select a small subset. RFE is an example of a wrapper approach (Solorio-Fernández, Carrasco-Ochoa & Martínez-Trinidad, 2020; Jenke, Peer & Buss, 2014).
- Embedded approaches combine feature selection and performance evaluation in a particular process, such as decision tree and linear discriminant analysis (LDA).
- Hybrid approaches comprise two phases: filtering to decrease the feature sizes, followed by wrapper to find the optimal subset of features from the remaining features. Hybrid approaches combine the advantages of the filter and wrapper approach to achieve a good trade-off between low computational overhead and increase accuracy in the associated classification task with the selected features (Cai et al., 2018; Solorio-Fernández, Carrasco-Ochoa & Martínez-Trinidad, 2020).

Class separability is an important evaluation criterion in the filter approach. It is determined by measuring the distance between classes, followed by the removal of features that lead to low separability values. Some research design evaluation measurements exist for determining the relevance between features and class (*e.g*., consistency and relevance measures, ranking and correlation measures, and dependency measures).

### Preference indices

On the basis of the literature reviews given in Aldayel, Ykhlef & Al-Nafjan (2020a), the following EEG indices were defined to measure consumers’ responses to marketing stimuli: approach/withdrawal (AW) index, valence, choice index, and effort index. AW index measures the frontal alpha asymmetry, indicating the motivation and desire as higher alpha activation of the left frontal cortex. Equation (10) is used to calculate the difference between the right and left PSD. Touchette’s AW index (Eq. 1) was also used to calculate AW scores by taking the difference between the right and left PSD, divided by their sum (using electrodes F4 and F3) (Touchette & Lee, 2017).

(1) $${\rm Touchette}\,{\rm AW}\,{\rm index} = \displaystyle{{alpha(F4) - alpha(F3)} \over {alpha(F4) + alpha(F3)}}$$

(2) $${\rm AW}\,{\rm index} = alpha(AF4,F4) - alpha(AF3,F3)$$

The valence indicates the asymmetrical activation of the frontal hemisphere. It is computed using the following four equations, which were well-explained in Al-Nafjan et al. (2017).

(3) $${\rm Vamv\_valence} = \displaystyle{{beta(AF3,F3)} \over {alpha(AF3,F3)}} - \displaystyle{{beta(AF4,F4)} \over {alpha(AF4,F4)}}$$

(4) $${\rm Kirk\_valence = ln[alpha(AF3,F3)]} - {\rm ln[alpha(AF4,F4)]}$$

(5) $${\rm Ram12\_valence = alpha(F4)} - {\rm beta(F3)}$$

(6) $${\rm Ram15\_valence = }\displaystyle{{{\rm alpha(F4)}} \over {{\rm beta(F4)}}} - \displaystyle{{{\rm alpha(F3)}} \over {{\rm beta(F3)}}}$$

The choice index measures choice possibility in decision-making from the frontal asymmetry of beta and gamma. Ramsoy’s equation (Ramsøy et al., 2018), as shown in Eq. (8), was used to calculate the choice index for each band individually (gamma and beta) using electrodes AF3 and AF4.

(7) $${\rm Choice}\,{\rm index(gamma)} = \displaystyle{{log(gamma(AF3)) - log(gamma(AF4))} \over {log(gamma(AF3)) + log(gamma(AF4))}}$$

(8) $${\rm Choice}\,{\rm index(beta)} = \displaystyle{{log(beta(AF3)) - logbeta((AF4))} \over {log(beta(AF3)) + log(beta(AF4))}}$$

The effort index indicates the cognitive and activity level of the theta in frontal cortex. The following equations were used to calculate the effort index:

(9) $${\rm Effort}\,{\rm Index\_1} = \displaystyle{{theta(F4) - theta(F3)} \over {theta(F4) + theta(F3)}}$$

(10) $${\rm Effort}\,{\rm Index\_2} = theta(AF4,F4) - theta(AF3,F3)$$

Moreover, a combination of preference indicators with different time—frequency analyses was used to measure EEG-based preference indices.

## Originality

Multiple studies have conducted BCI-based neuromarketing experiment to measure consumers’ reactions to product attractiveness (Touchette & Lee, 2017; You & Liu, 2020; Modica et al., 2018; Cartocci et al., 2017; Ramsøy et al., 2018; Panda, Chakladar & Dasgupta, 2021; Teo et al., 2018). Many computational approaches were used for the EEG-based preference detection such as fast Fourier transform, Hilbert–Huang spectrum, SVM, KNN, and quadratic discriminant analysis (Kim et al., 2015; Cohrdes et al., 2017; Teo et al., 2018; Vinay, Lerch & Leslie, 2021). To detect the user preferences for music. Other research used different marketing stimulus such as virtual 3D shapes or 3D rotating objects (Chew, Teo & Mountstephens, 2016; Teo, Hou & Mountstephens, 2017; Teo, Hou & Mountstephens, 2018).

Some modules of the consumer preference recognition system (Fig. 1) have been published earlier, including feature extraction and preference indices calculation (Aldayel, Ykhlef & Al-Nafjan, 2020a; Aldayel, Ykhlef & Al-Nafjan, 2021). In Aldayel, Ykhlef & Al-Nafjan (2020a), the initial experiment results with deep learning are reported to detect the EEG-based consumer preferences using the DEAP dataset and considering the PSD (one feature extraction technique) and valence features (one EEG index). In Aldayel, Ykhlef & Al-Nafjan (2021), the preference detection of neuromarketing dataset was examined using two feature extraction techniques: DWT and PSD. The effect of using combinations of the four EEG indices and different algorithms for feature extraction and classification, such as SVM, KNN, deep neural network (DNN), and random forest (RF), were primarily investigated. In Aldayel, Ykhlef & Al-Nafjan (2020b), the authors investigated EEG-based transfer learning and propose deep transfer learning models to transfer knowledge from emotion recognition to preference recognition to enhance the classification prediction accuracy.

This study highlights the need to use feature selections along with different classifiers to enhance the accuracy results of preference detection. A previous study used the same classification algorithm to measure how feature selection can improve the accuracy of preference detection (Aldayel, Ykhlef & Al-Nafjan, 2020a).

## Feature Selection Algorithms

In this study, several feature selection methods used for benchmark testing in previous BCI studies were chosen (Jiang et al., 2020; Jenke, Peer & Buss, 2014; Moon et al., 2013). These include mRMR, PCA, RFE, and ReliefF. Each feature selection algorithm used in this study is briefly explained in the following paragraphs.

### mRMR

Peng, Long & Ding (2005) proposed the mRMR filter method, which selects features on the basis of relevance and redundancy measures. This method initially involves the selection of features with the largest correlation of class label and the least correlation of other features. The statistical correlation is computed using mutual information I, thereby ensuring that the joint distribution can optimize the following two criteria simultaneously: (1) maximum relevance/dependence *D* between features and the class label $I\left( {{{\bf x}_a};{\bf y}} \right)$ and (2) minimum redundancy *R* of features $I\left( {{{\bf x}_a};{{\bf x}_b}} \right)$, which maximizes classification accuracy (Peng, Long & Ding, 2005; Jenke, Peer & Buss, 2014). Assuming a chosen set $\mathcal{P}_{q}$ of *q* features, the feature is selected by optimizing the combined criterion *D* − *R*:

(11) $$\mathop {\max }\limits_{{{\bf{x}}_a} \in {\cal X} - {{\cal P}_q}} \left[ {I\left( {{{\bf{x}}_a};{\bf{y}}} \right) - {1 \over q}\sum\limits_{{{\bf{x}}_b} \in {\rm{ }}{{\cal P}_q}} {I\left( {{{\bf{x}}_a};{{\bf{x}}_b}} \right)} } \right]$$

mRMR can be effectively integrated with wrappers to form a hybrid feature selection approach that can reduce computational overhead with greater speed and accuracy (Peng, Long & Ding, 2005; Jenke, Peer & Buss, 2014). Many researchers have found that mRMR is an effective feature selection approach for EEG signals (Atkinson & Campos, 2016; Moon et al., 2013; Jenke, Peer & Buss, 2014). mRMR-based feature selection improves the accuracy of the kernel classifier for emotion recognition. Moreover, another study of preference recognition showed that mRMR can enhance classifier accuracies with band power features (Moon et al., 2013).

### RFE

**Figure 3:**
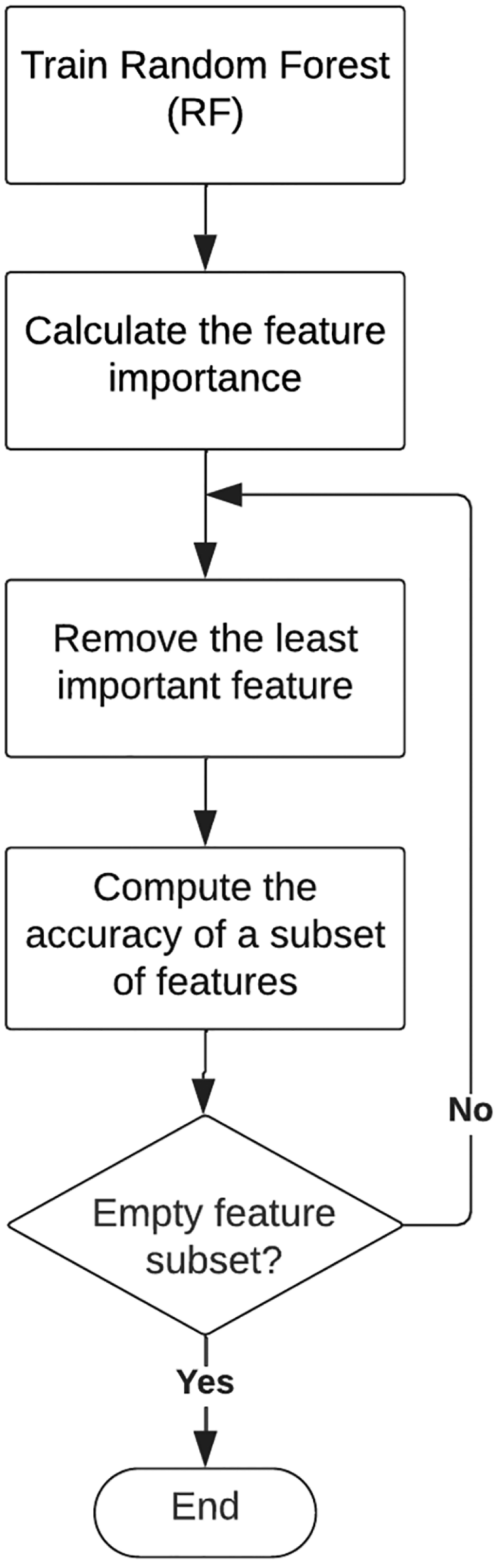
Flowchart of recursive feature elimination (RFE).

RFE is a wrapper method that is widely used for feature selection. It works by reducing features recursively and constructing a classification model on the remaining features. The algorithm rearranges the ranking of candidate feature subsets with corresponding classification accuracies for each iteration. The classification accuracy is used to select a combination of features that contributed the most to class label prediction (Cai et al., 2018; Jiang et al., 2020). RF classifier is used in the RF-RFE method to measure the importance of features and to build a classifier with high importance scores. Figure 3 illustrates the flowchart of RF-RFE 3 (Chen et al., 2018).

### ReliefF

Relief-based algorithms are widely used for their simplicity and efficiency. The ReliefF technique is a standard filtering approach for feature selection representing an extension of the original Relief algorithm. It is known as the best practice of Relief-based algorithms (Jenke, Peer & Buss, 2014). ReliefF assigns weights to each feature based on its ability to separate class labels. ReliefF relies on some neighbor’s k to specify the k-nearest hits and misses to avoid the sensitivity of feature dependencies. Multiple classes are considered to search the k-nearest elements that are weighted by their earlier probabilities. This change increases weight estimate reliability, particularly in noisy problems. The efficiency of ReliefF is attributed to the fact that it is “intended as a screener to identify a subset of features that cannot be the smallest and can still include some irrelevant and redundant features, but that is small enough to use with more refined approaches in a detailed analysis” (Urbanowicz et al., 2018; Jenke, Peer & Buss, 2014).

### PCA

Recently, unsupervised feature selection approaches have piqued research interest owing to their ability to select related features without depending on the class label. This makes them unbiased, allowing them to work effectively even in the absence of prior knowledge. Thus, they decrease the probability of overfitting (Solorio-Fernández, Carrasco-Ochoa & Martínez-Trinidad, 2020).

PCA is one of the most common unsupervised feature selection methods used for dimensionality reduction. It applies statistical methods to transform a set of interrelated measurements into a set of linearly unrelated principal components. The significance of PCA relates to its ability to reduce dimensionality without information loss while considering the complexity of signal extraction and classification. PCA extracts useful signals from the time series data of EEG signals. It compresses EEG signals into unrelated components for signal preprocessing and feature selection, thereby reducing the noise in the signal separation. In turn, EEG signals are reconstructed after separating noise. When PCA detects patterns in a signal, it can be imagined as involving the rotation of coordinate axes along with the combination of time points. The principal components are the components that have a maximum variance (Xie & Oniga, 2020; Torres et al., 2020).

## Methods

Three approaches were developed for supervised feature selection: RF-RFE, mRMR, and ReliefF. PCA was applied for unsupervised feature selections. Each algorithm was implemented using the Python programming language: RF-RFE and PCA with the scikit-learn package, mRMR with the pymrmr package, and ReliefF with the ReliefF package in the following hyperparameters:

- RF-RFE: Adjusted the number of selected features to 10.
- mRMR: Used the mutual information quotient to combine the relevance and redundancy measures.
- ReliefF: The number of neighbors was fixed as 10.
- PCA: The number of components was set to 1.

First, the 10 best combinations of features were selected using the RFE algorithm. In turn, the relationships between the preference indicators and class labels were measured using different measures, such as mRMR, feature importance, and ReliefF. Feature selection requires measurement and evaluation criteria, forming the basis for selecting a feature. The Bayesian error rate refers to the optimal measurement in classification problems. However, it is difficult to calculate the Bayesian error rate for each feature because it considers a combination of features in the calculation. Thus, researchers use other measures, such as correlation, dependency, and distance (Cai et al., 2018). Since the main goal of classification is to maximize predictive accuracy, several studies use accuracy as the primary measure. In this study, the accuracy before and after feature selection was measured *via* classification performance. The next subsections illustrate the dataset and feature selection approach used in this study, evaluate the importance of the selected features, and compare the classification performance before and after feature selection.

### Dataset

**Figure 4:**
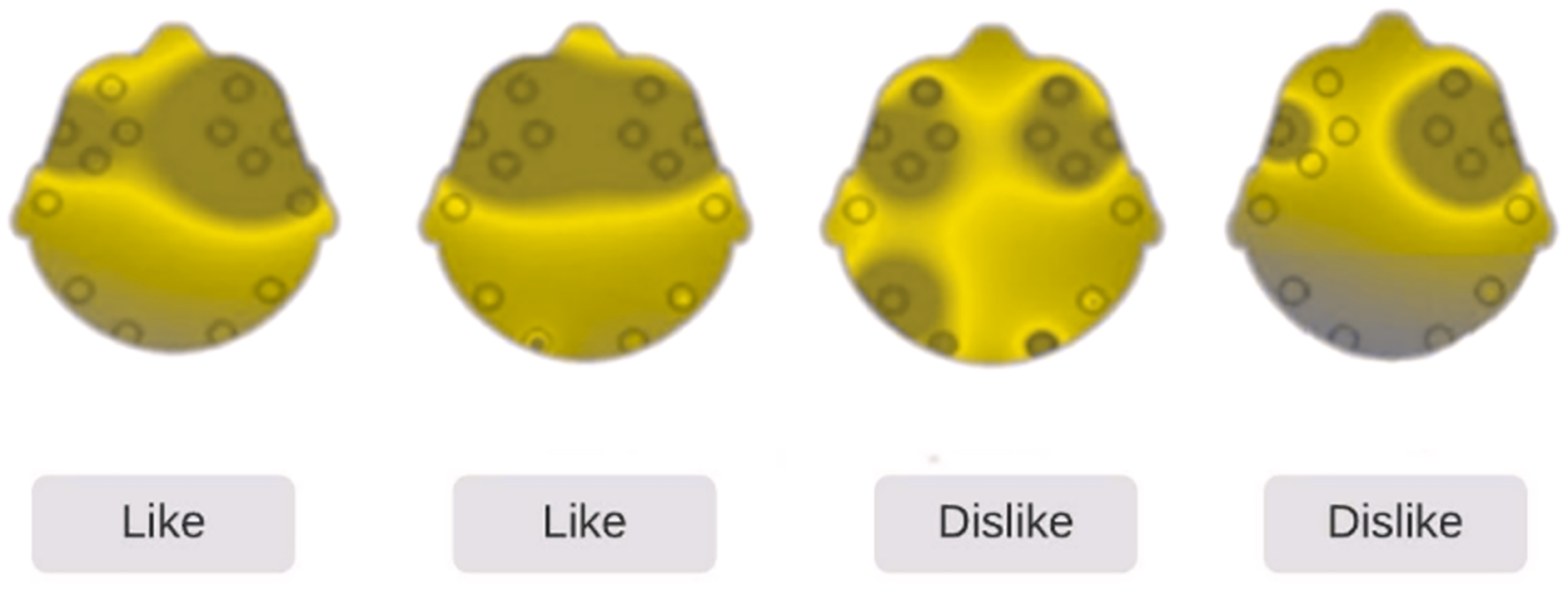
Brain activity map.

A public benchmark neuromarketing dataset (Yadava et al., 2017) that has been utilized in BCI-based neuromarketing experiments is used in this study. The Emotiv EPOC+ headset was used for capturing the EEG signals. A total of 25 male participants, aged 18–38 years, have recorded their 42 trials using the visual stimulus of 14 products, each having three variations (14 × 3 = 42). While the participants watched products on a computer screen, their EEG signals were recorded. Thus, 1,050 (= 42 × 25) records were logged for all participants. EEG signals were downsampled to 128 Hz and signal preprocessing (bandpass filter 4.0–45.0 Hz, independent component analysis, and a Savitzky–Golay filter) were performed to eliminate irrelevant features and artifacts. The features were collected from 14 electrodes located on “AF3, F7, F3, FC5, T7, P7, O1, O2, P8, T8, FC6, F4, F8, and AF4”. For preference recognition, four relevant electrodes were selected (F3, F4, AF3, and AF4). The selection of these electrodes was based on preference mapping with brain areas as explained in a previous research (Aldayel, Ykhlef & Al-Nafjan, 2020a). Responses in the form of either “like” or “dislike” were gathered from each participant watching a product, and EEG data were logged simultaneously. After each picture was displayed, the user’s preferred choice was recorded. The brain activity map in Fig. 4 shows the disruptions of brain activity in the two preference states “like” and “dislike”.

For validation, holdout validation was used to split the dataset: 80% for training and 20% for testing. For feature extraction, the fast Fourier transform method was used to extract PSD features. The dimensions of PSD features include 257 for each selected channel. Moreover, the sum and average of PSD feature across all four channels were combined. The average power of PSD was calculated using Morlet time—frequency transform into (61) features for each selected channel. In addition, (65) spectrogram Hanning features were extracted. The total numbers of PSD features were 2,367.

### Feature selection based on importance

**Figure 5:**
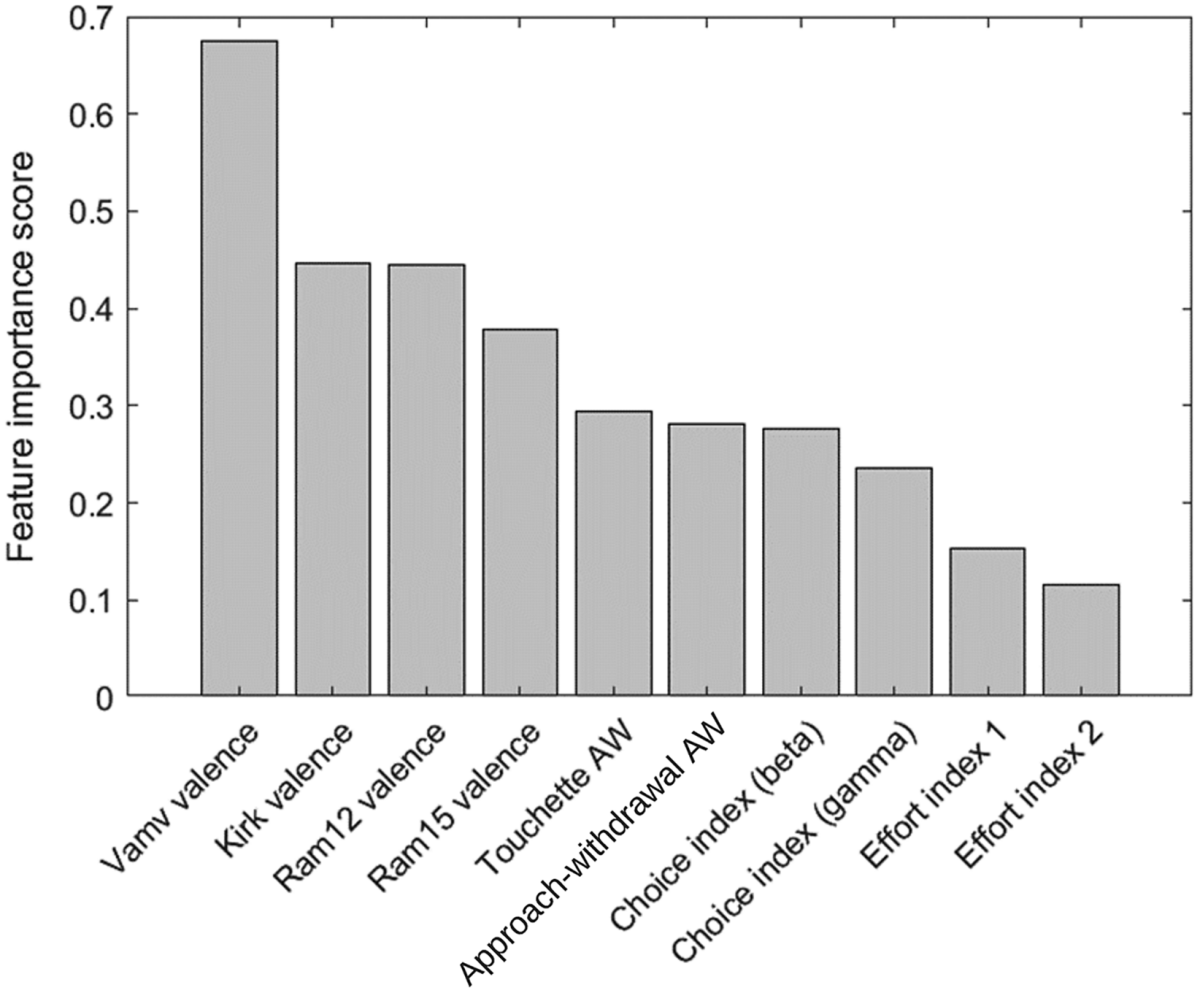
Top 10 feature rankings in mRMR.

**Figure 6:**
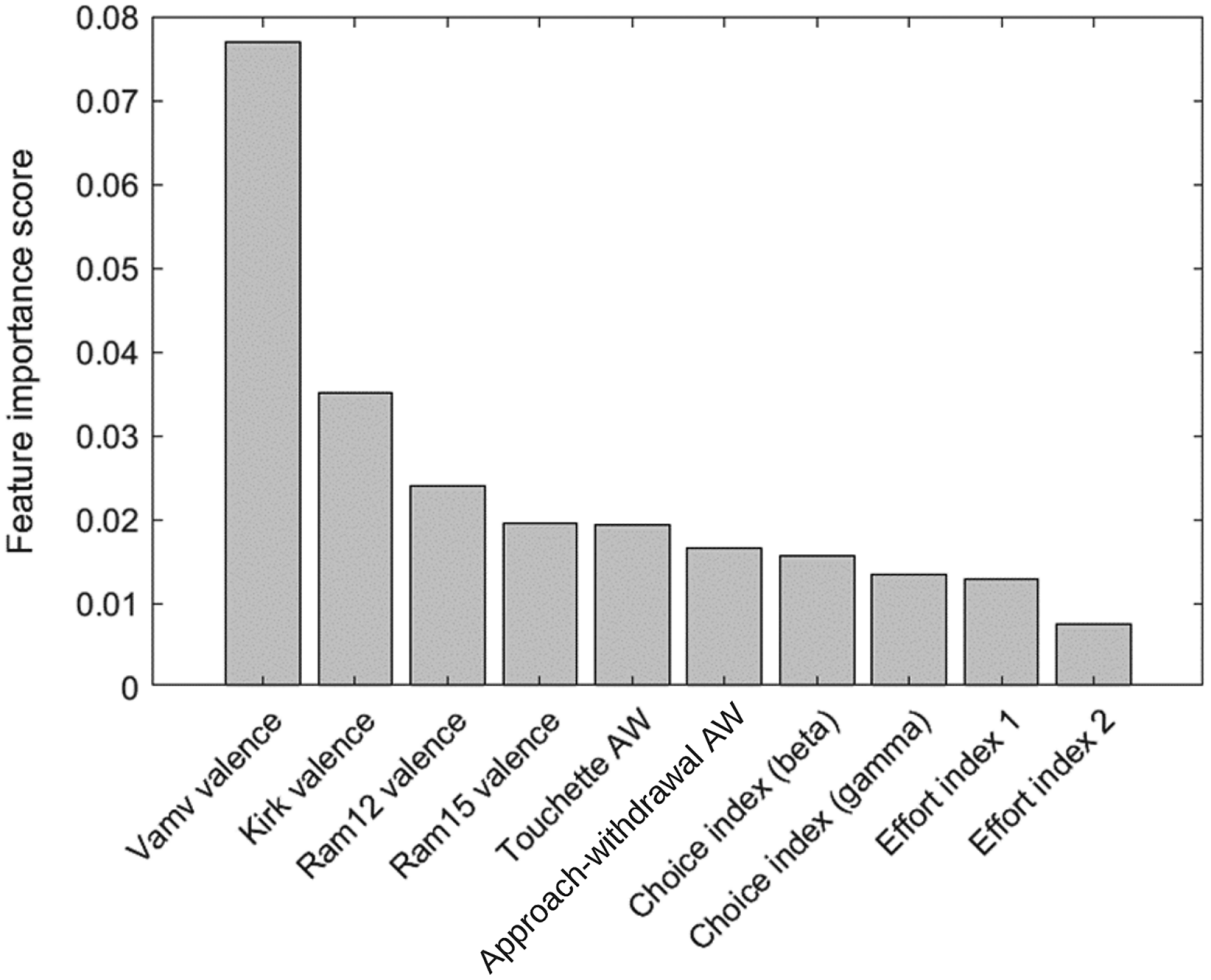
Top 10 feature rankings in ReliefF.

The top 10 features in RF-RFE were selected for their importance to the preference state were measured using RF, mRMR, and ReliefF. The mRMR and ReliefF algorithms were implemented using the fscmrmr and relieff functions in MATLAB. Moreover, the feature importance property in the RF algorithm implemented in the scikit-learn package in Python was used to calculate feature importance in RF-RFE. The variance between the top 10 most important features was indicated *via* the analysis of variance (ANOVA) *F*-test. Finally, the correlation between all of the top features and class labels was measured using a correlation matrix with a heatmap in Python’s seaborn package. Figures 5 and 6 show the top 10 feature rankings in mRMR and ReliefF, respectively.

### Feature selection and classification algorithms

This study aims to detect two preference states (“like” or “dislike”) in EEG neuromarketing data. Hence, intelligent classification algorithms were deployed to effectively mirror the preferences of the subjects. A DNN classifier is proposed and its performance is compared to the KNN, RF, LDA, and SVM classifiers. As such, four classifiers were used to discover the optimal preference index and a well-matched classifier marked with the highest accuracy: DNN, KNN, LDA, RF, and SVM. The default hyperparameters for the KNN, RF, and SVM algorithms were used in the scikit-learn package to adjust the following hyperparameters.

- KNN: Adjusted the number of neighbors to 1.
- RF: Adjusted the number of trees in the forest to 500, which were all processed in parallel.
- LDA: Set the number of components to 1.
- SVM: Used the kernel of RBF.

## Results

Neuroscience could reveal information about marketing-relevant behavior that is unobtainable through traditional methods, where neural activity can predict the preferences of consumer products. These insights aim to assist scholars and industry practitioners in uncovering the mechanisms underlying information processing by applying neuroimaging and computational approaches to understand the cognitive, neuronal, and emotional mechanisms related to marketing-relevant behavior and then applying them to different facets of marketing, which include marketing practice, product design, advertising, and branding (Garczarek-Bąk et al., 2021; Alvino et al., 2020). The next subsections illustrate the results of the twofold experiment: (1) the important results of different feature selection algorithms and (2) the classification results before and after feature selection.

### Feature importance results

**Figure 7:**
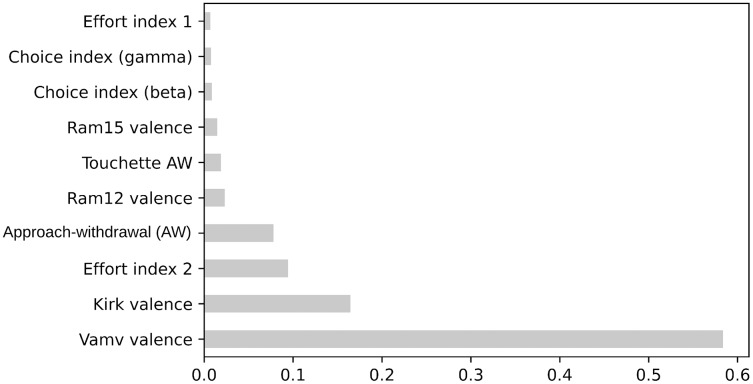
The *F*-values of top 10 features based on RF.

**Figure 8:**
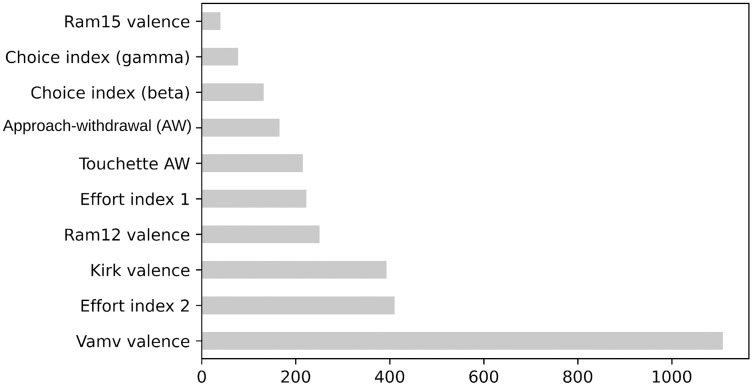
The *F*-values of top 10 features based on ANOVA.

Figures 5 and 6 present the top 10 feature rankings using the mRMR and ReliefF algorithms, respectively. Figure 7 presents the top 10 important features in the RF algorithm that were recursively considered in RFE. Since PCA separates components with the maximum variance; the ANOVA *F*-test illustrates the variance between the top 10 important features related to class labels (Fig. 8). The *F*-value scores check if the average for each combination of numerical features by the class label is significantly different.

**Figure 9:**
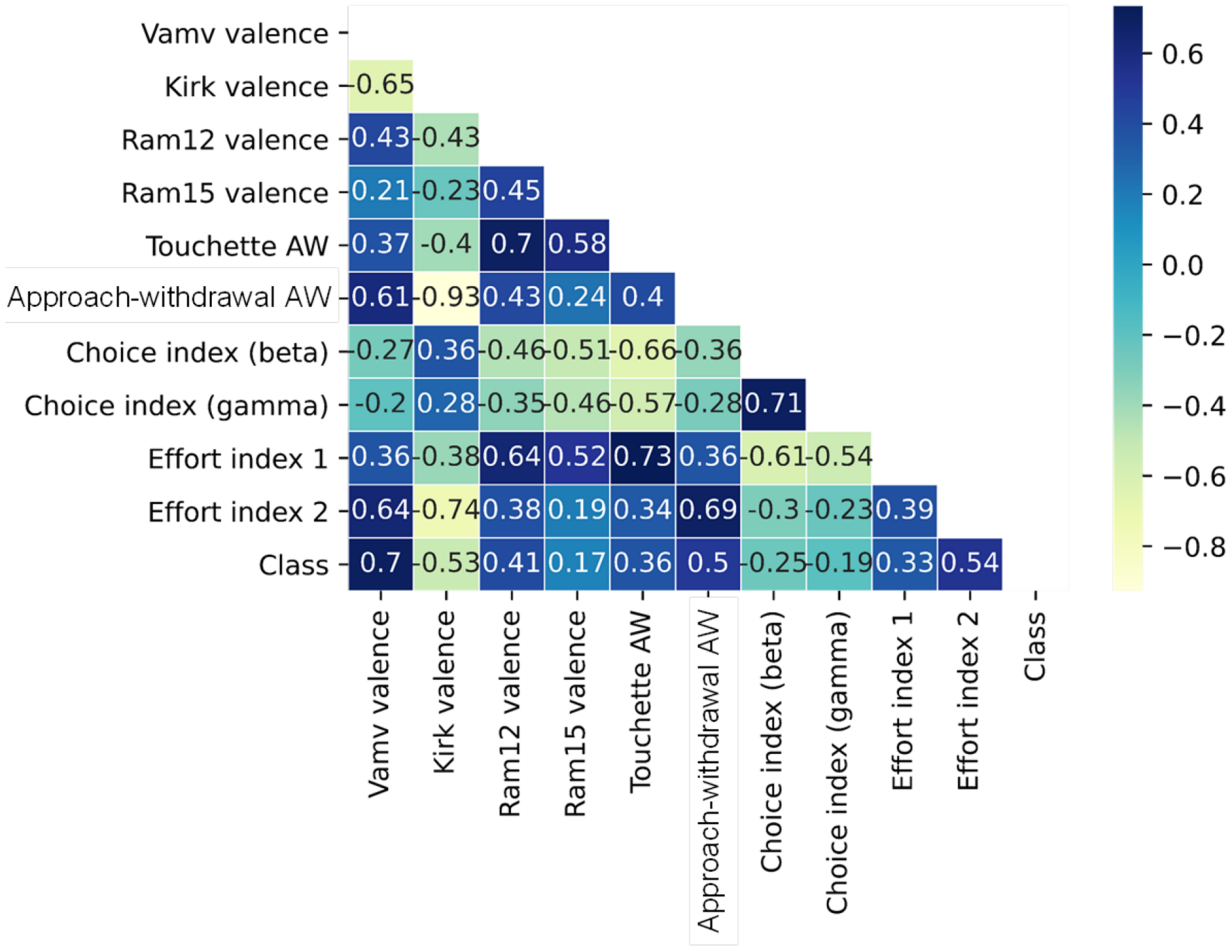
Correlation matrix with heatmap of top 10 features.

Correlation is a measure of the positive or negative linear relationship between any two features. A heatmap is used to illuminate the correlation between the top 10 important features. The correlation matrix in the heatmap (Fig. 9) illustrates the values of Kendall’s tau correlation coefficient between the top 10 important features and the class labels. Kendall’s rank correlation coefficient measures similarities of orderings of data that are ranked by their quantities. The most interesting features in terms of their correlation scores to the class label were Vamv valence (0.70), Kirk valence (0.53), AW (0.50), and effort index 2 (0.54). Notably, Vamv valence and Kirk valence were the top features in mRMR, ReliefF, RF, ANOVA F-measure, and Kendall’s rank correlation coefficients. The third most important feature was effort index 2 in RF, ANOVA F-measure, and Kendall’s rank correlation coefficient. This is consistent with the neuroscientific interpretation of the dataset owners (Yadava et al., 2017). They achieved the highest accuracy rate of 70.33% with the theta band, which was represented as effort index 2 in this research.

### Classification results

Since the results of feature selection depend on the classification performance, the classification performance before and after applying the feature selection was evaluated. The study aims to evaluate the effect of applying feature selection to enhance the identification of preference states (*i.e*., “like” and “dislike”) in the EEG neuromarketing data.

The EEG signal data was divided (80% training and 20% for testing). Classifiers that efficiently replicate the subjects’ preferences were used. The extracted features (PSD and preference indices) were fed into five classifiers (LDA, SVM, RF, DNN, and KNN).

**Figure 10:**
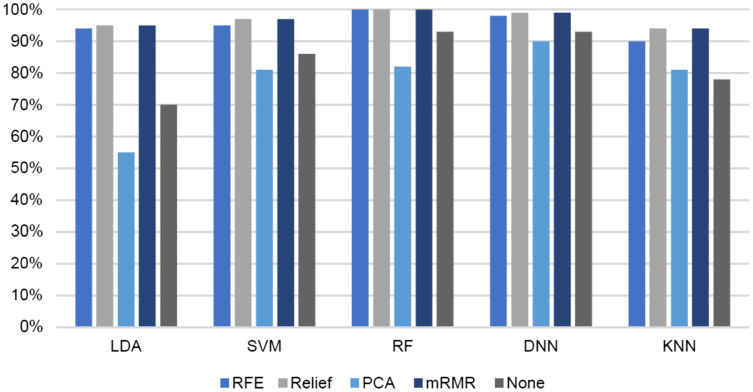
Effect of different feature selection algorithms (REF, ReliefF, PCA, and mRMR) on accuracy of different classifiers (LDA, SVM, RF, DNN and KNN).

**Figure 11:**
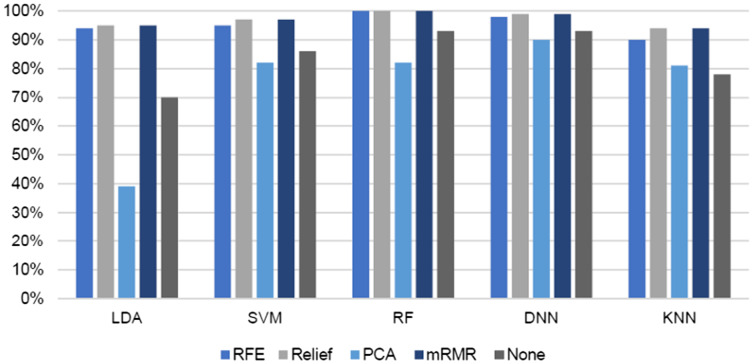
Effect of different feature selection algorithms (REF, ReliefF, PCA, and mRMR) on F1-measure of different classifiers (LDA, SVM, RF, DNN and KNN).

In comparison with other loss functions, the DNN result with the hinge loss function achieved the highest accuracy. The performances of these classifiers were compared with and without different feature selection methods (REF, ReliefF, PCA, feature importance, and mRMR), as presented in Table 1. Additionally, the number of selected features set to 10 in ReliefF, feature importance, and mRMR. In REF and feature importance, we also set the number of features to 30. In PCA, the features were reduced from 2,367 to 1,050. Figures 10 and 11 show the results from the viewpoint of feature selection. The figure demonstrates the effect of different feature selection algorithms on the accuracy and F-measure of the LDA, SVM, RF, DNN, and KNN classifiers.

**Table 1:** Classification results for different PSD-based feature selection methods: REF, ReliefF, PCA, and mRMR with different classifiers: DNN, KNN, RF, and SV.

Feature selection	Classifiers	Accuracy (%)	Recall (%)	Precision (%)	F-measure (%)
RFE	LDA	94	94	95	94
	SVM	95	95	95	95
	RF	100	100	100	100
	DNN	98	98	98	98
	KNN	90	90	90	90
ReliefF	LDA	95	95	95	95
	SVM	97	97	97	97
	RF	100	100	100	100
	DNN	99	99	99	99
	KNN	94	94	94	94
PCA	LDA	55	55	30	39
	SVM	81	81	83	82
	RF	82	82	84	83
	DNN	90	90	90	90
	KNN	81	81	82	81
mRMR	LDA	95	95	96	95
	SVM	97	97	97	97
	RF	100	100	100	100
	DNN	99	99	99	99
	KNN	94	94	94	94
Importance (10 features)	LDA	93	93	94	93
	SVM	98	98	98	98
	RF	100	100	100	100
	DNN	97	97	97	97
	KNN	94	94	94	94
Importance (30 features)	LDA	94	94	94	94
	SVM	96	96	96	96
	RF	97	97	97	97
	DNN	98	98	98	98
	KNN	93	93	93	93
None	LDA	70	70	71	70
	SVM	86	86	87	86
	RF	93	93	93	93
	DNN	93	93	93	93
	KNN	78	78	79	78

For all classifiers, the best results were achieved with mRMR and ReliefF. The KNN, LDA, and SVM classifiers yielded enhanced accuracies of 94%, 95%, and 97%, respectively, whereas DNN and RF achieved the highest accuracies of 99% and 100%, respectively. Similar results were achieved with the feature importance method, but all classifiers, except DNN, yielded better accuracies when the number of selected features was set to 10. DNN achieved a better accuracy at 98% with 30 features. The reason for this is that DNN works better with a large dataset. PCA did not yield enhanced accuracy compared with the other feature selection methods.

In summary, the mRMR feature selection algorithm achieved the best classification results (*i.e*., in terms of accuracy, recall, and precision) at 100% with RF and 99% with DNN (hinge function). RF reached the best results across all feature selection methods. The LDA, SVM, and KNN classifiers achieved an approximately 20% increase in accuracy after applying feature selection. Generally, feature selection was found to improve the performance of all classifiers.

## Discussion

From the ‘Results’ section, the comparison of accuracy and F-measure of different classifiers (LDA, SVM, RF, DNN, and KNN) after applying several feature selection algorithms (RF, ReliefF, PCA, feature importance, and mRMR) shows that the highest accuracies and F-measures were achieved with the DNN and RF classifiers at 99% and 100%, respectively. The LDA, KNN, and SVM classifiers improved noticeably in terms of their accuracy after applying feature selection. Specifically, LDA improved from 70% to 94%, SVM from 86% to 98%, and KNN from 78% to 94% The highest accuracy was 100% with RF, while the second-highest accuracy was 99% with DNN and the hinge loss function.

In summary, the best classification results (*i.e*., in terms of accuracy, recall, and precision) were achieved at 100% with RF and 99% with DNN (hinge function) using the mRMR feature selection algorithm. RF reached the best results across all feature selection methods. After feature selection, an increase in accuracy of approximately 20% was achieved for the LDA, SVM, and KNN classifiers. In general, feature selection was found to improve the performance of all classifiers.

Moreover, the result of this study was compared to other recent published studies that used the same dataset in their experiments but different feature extraction and classification techniques as shown in Table 2.

**Table 2:** A comparison between previous studies and the current study.

Ref.	Feature extraction	Classification	Accuracy (%)
Panda, Chakladar & Dasgupta (2021)	PSD	LSTM-based DNN	94.81
Yulita et al. (2020)	Wavelet-PSD	PAMELA-CL-based KNN	85
Alimardani & Kaba (2021)	PSD	CNN	74.57
Proposed method	PSD + mRMR feature selection	RF	100
		DNN	99

Panda, Chakladar & Dasgupta (2021) proposed a long short-term memory (LSTM)-based deep neural network model to classify the EEG dataset into four classes (most like, like, dislike, and most dislike). The PSD was used for feature extraction to extract the frequency bands. Their proposed model achieved 94.18% classification accuracy. They reported that their model achieved higher accuracy when compared with other computational methods, with an improvement of 11.71% and 3.24% when compared to SVM and RF, respectively.

Yulita et al. (2020) proposed a Partition Membership Based on Lazy Classifier (PAMELA-CL) based on KNN to classify EEG signals into two classes, namely, likes and dislikes. They used wavelets for feature extraction. They achieved 85% accuracy. They reported that PAMELA-CL achieves better performance (above 25%) compared to KNN.

Alimardani & Kaba (2021) proposed a deep convolutional neural network (CNN) for the classification of EEG signals to predict the relevant consumer preference from brain activity. The five frequency bands (delta, theta, alpha, beta, and gamma) were extracted, and the spectral energy associated with each band was calculated. The performance of a machine learning model comprising an ensemble of algorithms (SVM, RF, and logistic regression) was compared to the performance of a convolutional neural network (CNN). Their experimental results showed that the machine learning approach achieved an accuracy of 63.54% and an F1 score of 76.83%, whereas the proposed CNN model achieved an accuracy of 74.57% and an F1 score of 84.13%.

The comparison showed that the proposed method achieved higher accuracy result. For example, for the same number of classes, an improvement of 25.5% and 15% was achieved with the proposed method (mRMR for feature selection and RF classifier) when compared to Alimardani & Kaba (2021) and Yulita et al. (2020), respectively.

## Conclusions

Neuromarketing is an extension of the BCI field, which facilitates understanding and getting insights into the consumer perspective of personalized marketing. These insights are applied to measure and improve the effectiveness of different marketing contexts, such as in-store design, digital environment, advertising, product/service design and packaging, pricing, and branding. This study summarized the feature selection methods applied for neuromarketing preference detection systems. The importance of EEG indices to the preference state was measured, and the different computational intelligence methods for feature selection were explained. For selecting EEG features, four approaches were used: PCA, ReliefF, mRMR, and RFE. Feature selection for EEG signals was found to improve the performance of all classifiers.

Future directions for this work include considering building a large EEG dataset to fine-tune the model to improve the model’s performance. More alternative feature selection approaches, such as mutual information, least absolute shrinkage and selection operator, and stepwise linear discriminant analysis, could be explored to improve the detection results.

## Supplemental Information

10.7717/peerj-cs.944/supp-1 Supplemental Information 1 Code.

## Additional Information and Declarations

### Data Availability

The following information was supplied regarding data availability: The data is available at GitHub:
https://github.com/meagmohit/EEG-Datasets
